# Complete genome sequence of *Calditerrivibrio nitroreducens* type strain (Yu37-1^T^)

**DOI:** 10.4056/sigs.1523807

**Published:** 2011-02-20

**Authors:** Sam Pitluck, Johannes Sikorski, Ahmet Zeytun, Alla Lapidus, Matt Nolan, Susan Lucas, Nancy Hammon, Shweta Deshpande, Jan-Fang Cheng, Roxane Tapia, Cliff Han, Lynne Goodwin, Konstantinos Liolios, Ioanna Pagani, Natalia Ivanova, Konstantinos Mavromatis, Amrita Pati, Amy Chen, Krishna Palaniappan, Loren Hauser, Yun-Juan Chang, Cynthia D. Jeffries, John C. Detter, Evelyne Brambilla, Oliver Duplex Ngatchou Djao, Manfred Rohde, Stefan Spring, Markus Göker, Tanja Woyke, James Bristow, Jonathan A. Eisen, Victor Markowitz, Philip Hugenholtz, Nikos C. Kyrpides, Hans-Peter Klenk, Miriam Land

**Affiliations:** 1 DOE Joint Genome Institute, Walnut Creek, California, USA; 2 DSMZ - German Collection of Microorganisms and Cell Cultures GmbH, Braunschweig, Germany; 3 Los Alamos National Laboratory, Bioscience Division, Los Alamos, New Mexico, USA; 4 Biological Data Management and Technology Center, Lawrence Berkeley National Laboratory, Berkeley, California, USA; 5 Oak Ridge National Laboratory, Oak Ridge, Tennessee, USA; 6 HZI – Helmholtz Centre for Infection Research, Braunschweig, Germany; 7 University of California Davis Genome Center, Davis, California, USA; 8 Australian Centre for Ecogenomics, School of Chemistry and Molecular Biosciences, The University of Queensland, Brisbane, Australia

**Keywords:** moderately thermophilic, strictly anaerobic, motile, Gram-negative, chemoorganoheterotroph, hot spring, *Deferribacteraceae*, GEBA

## Abstract

*Calditerrivibrio nitroreducens* Iino *et al*. 2008 is the type species of the genus *Calditerrivibrio*. The species is of interest because of its important role in the nitrate cycle as nitrate reducer and for its isolated phylogenetic position in the Tree of Life. Here we describe the features of this organism, together with the complete genome sequence and annotation. This is the third complete genome sequence of a member of the family *Deferribacteraceae*. The 2,216,552 bp long genome with its 2,128 protein-coding and 50 RNA genes is a part of the *** G****enomic* *** E****ncyclopedia of* *** B****acteria and* *** A****rchaea * project.

## Introduction

Strain Yu37-1^T^ (= DSM 19672 = NBRC 101217) is the type strain of *Calditerrivibrio nitroreducens* which in turn is the type and sole species of the genus *Calditerrivibrio* [1]. The genus *Calditerrivibrio* is one out of six genera in the family *Deferribacteraceae* [2-6]. The genus name is derived from Latin adjective “*caldus*”, hot, “*terra*”, the earth, and “*vibrio*”, a vibrio, referring to a vibroid shaped bacterium in a hot terrestrial environment. The species epithet *nitroreducens* derives from the Greek name “*nitron*”, nitrite, nitrate, and “*reducens*”, drawing backwards, referring to its nitrate-reducing physiology [1]. Strain Yu37-1^T^ was isolated from hot-spring water from Yumata, Nagano, Japan. No further cultivated strains belonging to the species *C. nitroreducens* have been described so far. Here we present a summary classification and a set of features for *C. nitroreducens* strain Yu37-1^T^, together with the description of the complete genomic sequencing and annotation.

## Classification and features

A representative genomic 16S rRNA sequence of strain Yu37-1^T^ was compared using BLAST under default settings (e.g., considering only only the high-scoring segment pairs (HSPs) from the best 250 hits) with the most recent release of the Greengenes database [7] and the relative frequencies of taxa and keywords (reduced to their stem [8]) were determined, weighted by BLAST scores. The most frequently occurring genera were *Deferribacter* (33.4%), *Alteromonas* (21.3%), *Magnetococcus* (9.4%), *Shuttleworthia* (7.5%) and *Geovibrio* (7.3%) (61 hits in total). Regarding the single hit to sequences from members of the species, the average identity within HSPs was 99.9%, whereas the average coverage by HSPs was 96.7%. Among all other species, the one yielding the highest score was *Deferribacter desulfuricans*, which corresponded to an identity of 88.1% and an HSP coverage of 86.0%. The highest-scoring environmental sequence was DQ424925 ('Enrichment and Thermophilic Mediator-Less Microbial Fuel Cell thermophilic microbial fuel cell enriched artificial wastewater clone 1B62') [9], which showed an identity of 99.7% and an HSP coverage of 98.2%. The most frequently occurring keywords within the labels of environmental samples were 'microbiota' (4.1%), 'microbi' (4.1%), 'intestin' (4.0%), 'mous' (3.8%) and 'compet, exploit, inflamm, salmonella, typhimurium' (3.7%) (183 hits in total). The most frequently occurring keywords within the labels of environmental samples which yielded hits of a higher score than the highest scoring species were 'microbi' (8.6%), 'thermophil' (6.7%), 'enrich' (5.7%), 'cell, fuel' (5.3%) and 'spring' (3.6%) (21 hits in total), which seem to fit to the features known for *C. nitroreducens*.

**Figure 1:**
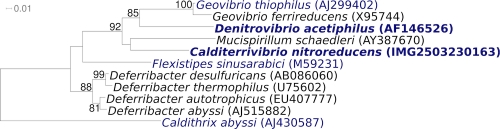
Phylogenetic tree highlighting the position of *C. nitroreducens* Yu37-1^T^ relative to the other type strains within the family *Deferribacteraceae*. The tree was inferred from 1,470 aligned characters [10,11] of the 16S rRNA gene sequence under the maximum likelihood criterion [12] and rooted in accordance with the current taxonomy. The branches are scaled in terms of the expected number of substitutions per site. Numbers above branches are support values from 1,000 bootstrap replicates [13] if larger than 60%. Lineages with type strain genome sequencing projects registered in GOLD [14] are shown in blue, published genomes in bold [15].

Figure 1 shows the phylogenetic neighborhood of *C. nitroreducens* Yu37-1^T^ in a 16S rRNA based tree. The two copies of the 16S rRNA gene in the genome differ by one nucleotide from each other any by up to one nucleotide from the previously published 16S rRNA sequence (AB364234).

**Figure 2:**
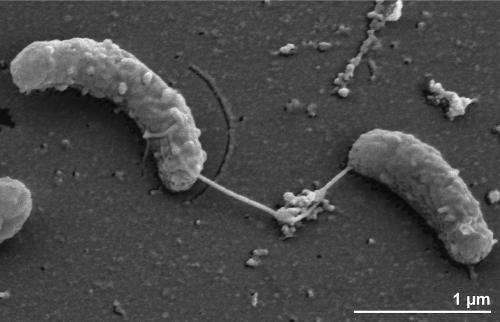
Scanning electron micrograph of *C. nitroreducens* Yu37-1^T^

Cells of the strain Yu37-1^T^ are vibrio-shaped, 0.4-0.5 x 1.4-2.0 µm in size, occur singly or in pairs and stain Gram-negative [1] (Table 1 and Figure 2). No spore formation was detected for Yu37-1^T^ [1]. No data is available on the generation time of strain Yu37-1^T^. Nitrate is the only electron acceptor utilized, with ammonium as the end product [1]. Elemental sulfur, sulfate, sulfite, nitrite, iron (III) oxide, manganese (IV) oxide, selenate, selenite, arsenate, arsenite, fumarate and oxygen are not used as alternative electron acceptors [1]. Acetate, pyruvate, lactate, fumarate, succinate, malate, yeast extract, peptone and Casamino acids are utilized as electron donors with nitrate as the electron acceptor; fermentative growth has not been observed [1]. Strain Yu37-1^T^ is strictly anaerobic and catalase negative [1].

**Table 1:** Classification and general features of *C. nitroreducens* Yu37-1^T^ according to the MIGS recommendations [16]

**MIGS ID**	**Property**	**Term**	**Evidence code**
	Current classification	Domain *Bacteria*	TAS [17]
		Phylum *Deferribacteres*	TAS [18-20]
		Class *Deferribacteres*	TAS [18,21]
		Order *Deferribacterales*	TAS [18,22]
		Family *Deferribacteraceae*	TAS [18,23]
		Genus *Calditerrivibrio*	TAS [1]
		Species *Calditerrivibrio nitroreducens*	TAS [1]
		Type strain Yu37-1	TAS [1]
	Gram stain	negative	TAS [1]
	Cell shape	vibrio-shaped	TAS [1]
	Motility	motile, single polar flagellum	TAS [1]
	Sporulation	none	TAS [1]
	Temperature range	30°C–65°C	TAS [1]
	Optimum temperature	55°C	TAS [1]
	Salinity	< 0.5% NaCl	TAS [1]
MIGS-22	Oxygen requirement	strictly anaerobic	TAS [1]
	Carbon source	carbohydrates	TAS [1]
	Energy source	chemoorganoheterotrophic	TAS [1]
MIGS-6	Habitat	hot spring	TAS [1]
MIGS-15	Biotic relationship	not reported	
MIGS-14	Pathogenicity	not reported	
	Biosafety level	1	TAS [24]
	Isolation	hot spring water	TAS [1]
MIGS-4	Geographic location	Yumata, Nagano, Japan	TAS [1]
MIGS-5	Sample collection time	2008 or before	TAS [1]
MIGS-4.1 MIGS-4.2	Latitude Longitude	36.83 138.22	TAS [1]
MIGS-4.3	Depth	0 m, surface waters	TAS [1]
MIGS-4.4	Altitude	not reported	

Evidence codes - IDA: Inferred from Direct Assay (first time in publication); TAS: Traceable Author Statement (i.e., a direct report exists in the literature); NAS: Non-traceable Author Statement (i.e., not directly observed for the living, isolated sample, but based on a generally accepted property for the species, or anecdotal evidence). These evidence codes are from of the Gene Ontology project [25]. If the evidence code is IDA, then the property was directly observed by one of the authors or an expert mentioned in the acknowledgements.

### Chemotaxonomy

The predominant compounds in whole cell lipids of *C. nitroreducens* strain Yu37-1^T^ are saturated branched-chain fatty acids: iso-C_14:0_ (26.3%), anteiso-C_15:0_ (24.1%), iso-C_13:0_ (7.7%), C_18:0_ (7.2%), C_16:0_ (6.2%), iso-C_16:0_ (5.7%) and anteiso-C_13:0_ (5.3%) [1]. Menaquinone MK-8 was identified as the major quinone [1].

## Genome sequencing and annotation

### Genome project history

This organism was selected for sequencing on the basis of its phylogenetic position [26], and is part of the *** G****enomic* *** E****ncyclopedia of* *** B****acteria and* *** A****rchaea * project [27]. The genome project is deposited in the Genomes On Line Database [14] and the complete genome sequence is deposited in GenBank. Sequencing, finishing and annotation were performed by the DOE Joint Genome Institute (JGI). A summary of the project information is shown in Table 2.

**Table 2:** Genome sequencing project information

**MIGS ID**	**Property**	**Term**
MIGS-31	Finishing quality	Finished
MIGS-28	Libraries used	Tree genomic libraries: one 454 pyrosequence standard library, one 454 PE library (7 kb insert size), one Illumina library
MIGS-29	Sequencing platforms	Illumina GAii, 454 GS FLX Titanium
MIGS-31.2	Sequencing coverage	150.7 × Illumina; 68.8 × pyrosequence
MIGS-30	Assemblers	Newbler version 2.5-internal-10Apr08-1-threads, Velvet, phrap
MIGS-32	Gene calling method	Prodigal 1.4, GenePRIMP
	INSDC ID	CP002347 (chromosome) CP002348 (plasmid)
	Genbank Date of Release	December 7, 2010
	GOLD ID	Gc01554
	NCBI project ID	49523
	Database: IMG-GEBA	2503707001
MIGS-13	Source material identifier	DSM 19672
	Project relevance	Tree of Life, GEBA

### Growth conditions and DNA isolation

*C. nitroreducens* Yu37-1^T^, DSM 19672, was grown anaerobically in DSMZ medium 1112 (Calditerrivibrio medium) [28] at 55°C. DNA was isolated from 0.5-1 g of cell paste using Qiagen Genomic 500 DNA Kit (Qiagen, Hilden, Germany) following the standard protocol as recommended by the manufacturer, with modification st/DL for cell lysis as described in [27]. DNA is available through the DNA Bank Network [29].

### Genome sequencing and assembly

The genome was sequenced using a combination of Illumina and 454 sequencing platforms. All general aspects of library construction and sequencing can be found at the JGI website [30]. Pyrosequencing reads were assembled using the Newbler assembler (Table 2). The initial Newbler assembly, consisting of seven contigs in four scaffolds, was converted into a phrap assembly [31] by making fake reads from the consensus to collect the read pairs in the 454 paired end library. Illumina GAii sequencing data (334.0 Mb) was assembled with Velvet [32] and the consensus sequences were shredded into 1.5 kb overlapped fake reads and assembled together with the 454 data. The 454 draft assembly was based on 152.9 Mb of 454 draft data and all of the 454 paired end data. Newbler parameters are -consed -a 50 -l 350 -g -m -ml 20. The Phred/Phrap/Consed software package [31] was used for sequence assembly and quality assessment in the subsequent finishing process. After the shotgun stage, reads were assembled with parallel phrap (High Performance Software, LLC). Possible mis-assemblies were corrected with gapResolution [30], Dupfinisher [33], or sequencing cloned bridging PCR fragments with subcloning or transposon bombing (Epicentre Biotechnologies, Madison, WI). Gaps between contigs were closed by editing in Consed, by PCR and by Bubble PCR primer walks (J.-F.Chang, unpublished). A total of 24 additional reactions were necessary to close gaps and to raise the quality of the finished sequence. Illumina reads were also used to correct potential base errors and increase consensus quality using a software Polisher developed at JGI [34]. The error rate of the completed genome sequence is less than 1 in 100,000. Together, the combination of the Illumina and 454 sequencing platforms provided 219.5 × coverage of the genome. The final assembly contained 438,623 pyrosequence and 43,957,307 Illumina reads.

### Genome annotation

Genes were identified using Prodigal [35] as part of the Oak Ridge National Laboratory genome annotation pipeline, followed by a round of manual curation using the JGI GenePRIMP pipeline [36]. The predicted CDSs were translated and used to search the National Center for Biotechnology Information (NCBI) nonredundant database, UniProt, TIGRFam, Pfam, PRIAM, KEGG, COG, and InterPro databases. Additional gene prediction analysis and functional annotation was performed within the Integrated Microbial Genomes - Expert Review (IMG-ER) platform [37].

## Genome properties

The genome consists of a 2,157,835 bp long chromosome with a 36% GC content and a 58,717 bp plasmid with 31% GC content (Table 3 and Figures 3a and 3b). Of the 2,278 genes predicted, 2,128 were protein-coding genes, and 50 RNAs; 27 pseudogenes were identified. The majority of the protein-coding genes (76.5%) were assigned with a putative function while the remaining ones were annotated as hypothetical proteins. The distribution of genes into COGs functional categories is presented in Table 4.

**Table 3:** Genome Statistics

**Attribute**	Value	% of Total
Genome size (bp)	2,216,552	100.00%
DNA coding region (bp)	2,076,059	93.66%
DNA G+C content (bp)	789,723	35.63%
Number of replicons	2	
Extrachromosomal elements	1	
Total genes	2,178	100.00%
RNA genes	50	2.30%
rRNA operons	2	
Protein-coding genes	2,128	97.70%
Pseudo genes	27	1.24%
Genes with function prediction	1,666	76.49%
Genes in paralog clusters	190	8.72%
Genes assigned to COGs	1,731	79.48%
Genes assigned Pfam domains	1,800	82.64%
Genes with signal peptides	295	13.54%
Genes with transmembrane helices	520	23.88%
CRISPR repeats	3	

**Figure 3a:**
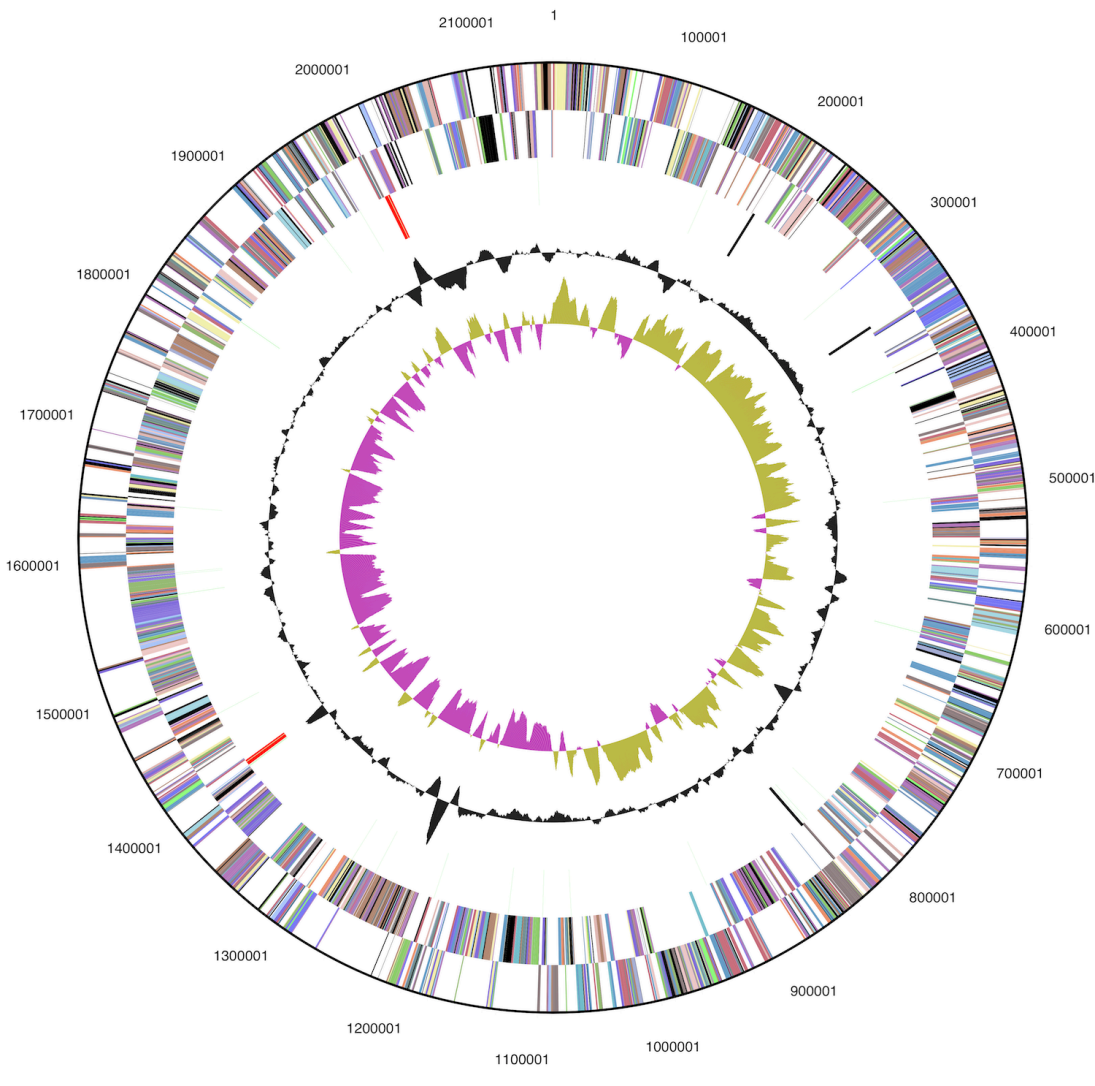
Graphical circular map of the chromosome. From outside to the center: Genes on forward strand (color by COG categories), Genes on reverse strand (color by COG categories), RNA genes (tRNAs green, rRNAs red, other RNAs black), GC content, GC skew.

**Figure 3b:**
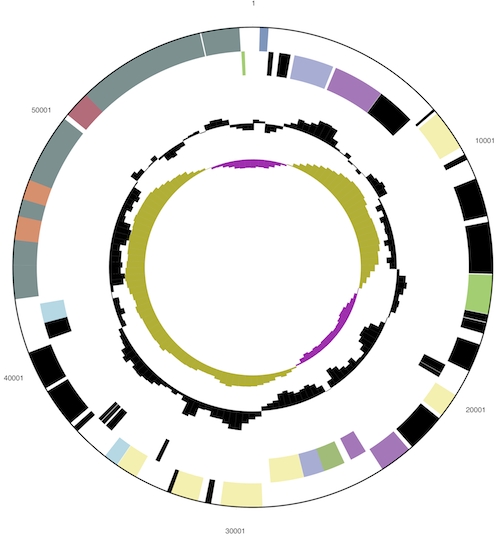
Graphical circular map of the plasmid (not drown to scale with chromosome). From outside to the center: Genes on forward strand (color by COG categories), Genes on reverse strand (color by COG categories), RNA genes (tRNAs green, rRNAs red, other RNAs black), GC content, GC skew.

**Table 4:** Number of genes associated with the general COG functional categories

Code	value	%age	Description
J	141	7.4	Translation, ribosomal structure and biogenesis
A	0	0.0	RNA processing and modification
K	78	4.1	Transcription
L	96	5.0	Replication, recombination and repair
B	1	0.1	Chromatin structure and dynamics
D	22	1.2	Cell cycle control, cell division, chromosome partitioning
Y	0	0.0	Nuclear structure
V	34	1.8	Defense mechanisms
T	139	7.3	Signal transduction mechanisms
M	145	7.6	Cell wall/membrane/envelope biogenesis
N	81	4.3	Cell motility
Z	0	0.0	Cytoskeleton
W	0	0.0	Extracellular structures
U	68	3.6	Intracellular trafficking and secretion, and vesicular transport
O	84	4.4	Posttranslational modification, protein turnover, chaperones
C	145	7.6	Energy production and conversion
G	67	3.5	Carbohydrate transport and metabolism
E	142	7.5	Amino acid transport and metabolism
F	53	2.8	Nucleotide transport and metabolism
H	112	5.9	Coenzyme transport and metabolism
I	58	3.0	Lipid transport and metabolism
P	87	4.6	Inorganic ion transport and metabolism
Q	25	1.3	Secondary metabolites biosynthesis, transport and catabolism
R	214	11.2	General function prediction only
S	113	5.9	Function unknown
-	447	20.5	Not in COGs
